# Dysregulation of SRF-Regulated Genes Accelerates the Development of Myocardial Aging

**DOI:** 10.1093/geroni/igaf122.2539

**Published:** 2025-12-31

**Authors:** Xiaomin Zhang, Gohar Azhar, Sayed Hasan, Jeanne Wei

**Affiliations:** University of Arkansas for Medical Sciences, Little Rock, Arkansas, United States; University of Arkansas for Medical Sciences, Little Rock, AR, LITTLE ROCK, Arkansas, United States; University of Arkansas for Medical Sciences, Little Rock, Arkansas, United States; University of Arkansas for Medical Sciences, Little Rock, AR, LITTLE ROCK, Arkansas, United States

## Abstract

Background Myocardial aging is associated with structural and functional changes that increase susceptibility to heart failure. Serum response factor (SRF), a key transcription factor regulating cytoskeletal, metabolic, and stress-response genes, plays a critical role in cardiac homeostasis. An increased expression of SRF has been observed in cardiac hypertrophy and aging. This study aims to investigate the impact of SRF dysregulation on myocardial aging. Methods We generated a transgenic mouse model (Tg) with the mild overexpression of the SRF gene in the heart. The echocardiographic and histological analyses demonstrated that the 6-month-old Tg mice manifested the phenotype of cardiac hypertrophy and aging. We analyzed the cardiac gene expression profile in Tg vs Non-Tg. Quantitative PCR and Western blotting confirmed gene expression. Bioinformatics tools were used to analyze SRF binding sites (CArG and CArG-like motifs) and categorize affected pathways. Results and conclusions Over 200 SRF-regulated genes exhibited significant changes in the Tg versus non-Tg mouse hearts (P < 0.05). These genes are involved in energy metabolism, lipid turnover, cardiac muscle contraction, transcriptional and translational regulation, and stress responses. Notably, the genes critical for fatty acid metabolism, lipid transport and mitochondrial function were downregulated, whereas genes involved in glycolysis were upregulated. Taken together, dysregulation of SRF-regulated genes triggers “metabolic reprogramming” leading to reduced fatty acid metabolism and mitochondrial function, as well as increased glycolysis. These changes ultimately decrease the cardiac metabolism efficiency and cardiac function. Therefore, SRF is a potential therapeutic target for improving cardiac metabolism and function in aging and pathological conditions.

